# Supplementary material for: Linking soil biology and chemistry in biological soil crust using isolate exometabolomics
Source: Nat Commun. 2018 Jan 2;9:19. doi: 10.1038/s41467-017-02356-9 (PMC5750228; doi:10.1038/s41467-017-02356-9)
Supplement: Supplementary file 1 — Supplementary Information [file 41467_2017_2356_MOESM1_ESM.pdf]

SUPPLEMENTARY METHODS

Metagenome library construction, sequencing, quality control, trimming, and filtering

Metagenomic library preparation was done by the Functional Genomics Laboratory at UC Berkeley using the Covaris shearing kit (Woburn, MA) and the IntegenX Apollo 324 robot (WaferGen Bio-systems, Inc, Fremont, CA). Samples were sequenced at QB3 (UC Berkeley, Berkeley, CA) using an Illumina HiSeq4000 (San Diego, CA, USA) to obtain paired-end, 150-bp with 400-bp inserts. The quality score profile of each sample was checked using FastQC, and based on those results, each sequence was trimmed 10 bp from the 5' end and 15 bp from the 3' end to remove regions with sequencing biases. The mean Q score for all trimmed sequences was > 30.

Low-complexity sequences were removed using the *dust* approach with the percent ambiguous bases set to <50% and maximum allowed *dust* score set to 50 using prinseq (http://sourceforge.net/projects/prinseq/files/standalone/). The orphan reads (i.e., with only one pair passing the filtering process) were removed using an in-house script. The total amount of sequence that were put into the metagenome assembly were as follows:

Stage	Time Point	Gb(post QC)
early	3 min	14.048
earlymid	3 min	13.43
latemid	3 min	13.482
late	3 min	14.57
early	9 h	13.024
earlymid	9 h	14.33
latemid	9 h	13.3
late	9 h	11.73
early	18 h	14.428
earlymid	18 h	14.262
latemid	18 h	14.1
late	18 h	12.118
early	42 h	13.468
earlymid	42 h	17.172
latemid	42 h	11.504
late	42 h	14.122
early	49.5 h	13.48
earlymid	49.5 h	11.822
latemid	49.5 h	14.29
late	49.5 h	12.328

Metagenome co-assembly

Trimmed and filtered sequences from all samples were concatenated into a single fastq file, giving a total of 395 Gb of sequence from 28 samples. These sequences were coassembled using MEGAHIT with the following parameters: k-list=27,35,41,51,71,81,91,99,109 and min-count=2 (all other parameters were left at default). The co-assembly required 216 hours on a 2000 GB memory instance on NERSC's genepool cluster. The mapping of reads to the scaffolds using Bowtie 2[1], the post processing of sequence alignments using samtools[2] and customized R and python scripts. The final assembly was in 163345 scaffolds (>1Kb) with an N50 size of 3.5Kb and had a total length of 0.63 Gb. The

largest scaffold length was 0.6 Mb. All of final scaffolds we tested for the presence of chimeric assemblies using paired-end read and depth-of-coverage consistency with in-house scripts.

Binning, bin evaluation and curation

The final set of quality controlled scaffolds were binned into genome bins using a Gaussian Mixture Model (GMM) clustering using tetranucleotide frequencies and depth of coverage across 28 samples as features. The depth of coverage profiles for the scaffolds were estimated based on mapping of reads from each sample to the scaffolds using Bowtie 2 (with options --sensitive, max# of mismatches N=1) and calculating the mean coverage for each scaffold from each of the samples. Tetranucleotide frequencies were calculated using in-house scripts. GMM clustering was performed using CONCOCT[3] with number of clusters set to 500 (-c 500) to obtain preliminary bins.

Bin completion and purity was evaluated using a set of single-copy ribosomal proteins. Scaffolds having 17 single-copy genes encoding ribosomal proteins (*rplB, rplC, rplD, rplE, rplF, rplJ, rplN, rplO, rplP, rplR, rplV, rplX, rpsC, rpsH, rpsJ, rpsQ, rpsS*) were determined based on the scoring by the corresponding TIGRFAM[4] and Pfam[5] models. Preliminary bins were checked for over- and under-binning and manually curated using DNA depth of coverage, GC content, and complementarity of the single-copy ribosomal gene set.

Functional Annotation

Open reading frames (ORFs) were predicted from the final set of scaffolds using Prodigal's[6] meta procedure (-p meta). Sequence similarity searches of protein sequences from the predicted ORFs were performed using USEARCH[7] (-ublast -query_cov 0.5 –target_cov 0.5 –id 0.97) against UniRef100[8]. Protein domain annotations were predicted with InterProScan[9]. Non-coding RNAs were predicted using Infernal (with cmsearch using default options against Rfam 11[10].

SUPPLEMENTARY FIGURES

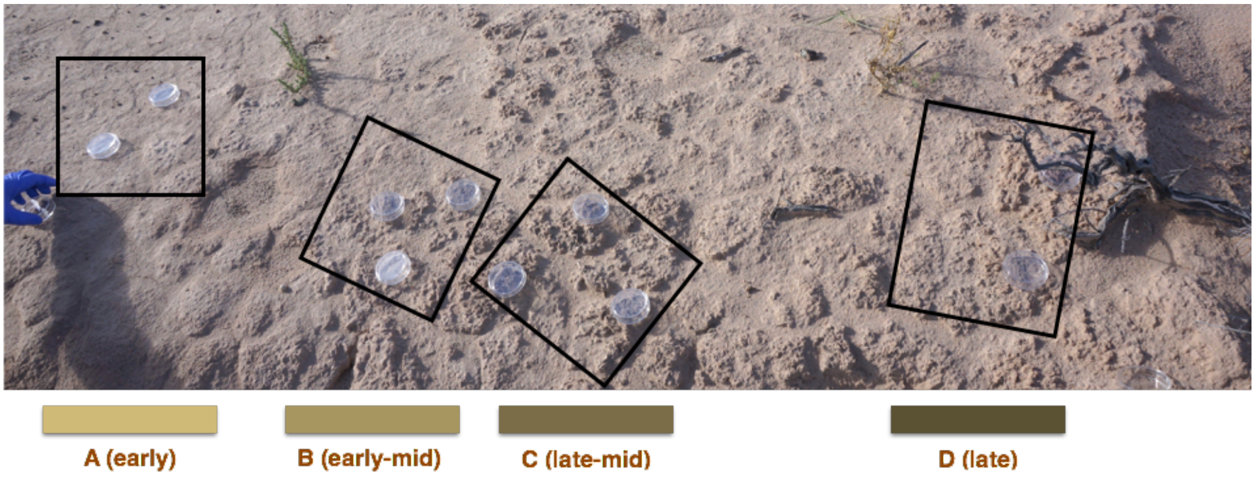

Supplementary Figure 1. Biocrust developmental transect. Four (apparent) successional stages of non-mossy Cyanobacteria biocrust were collected and ranged from early (level A) to late (level D).

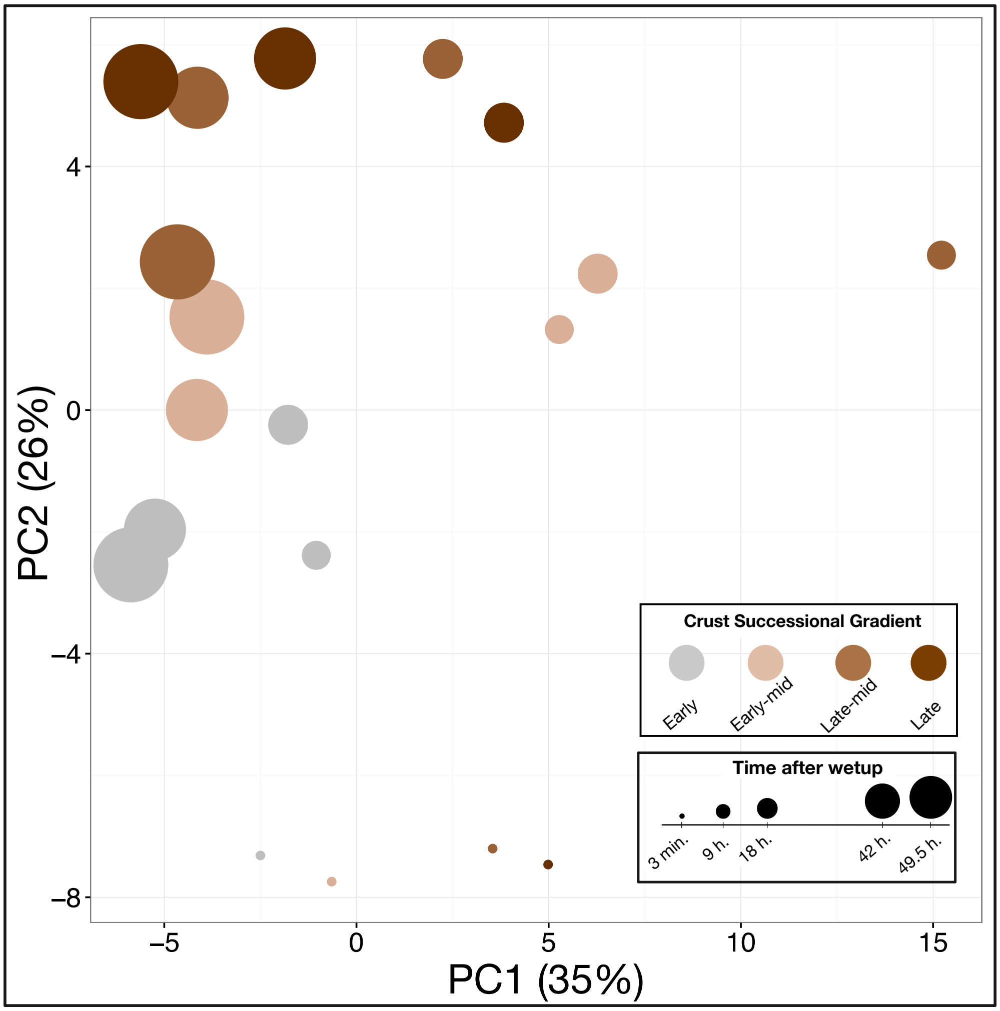

Supplementary Figure 2. PCA biplot of samples based on metabolite abundance (n=2-5 for each point)**.**

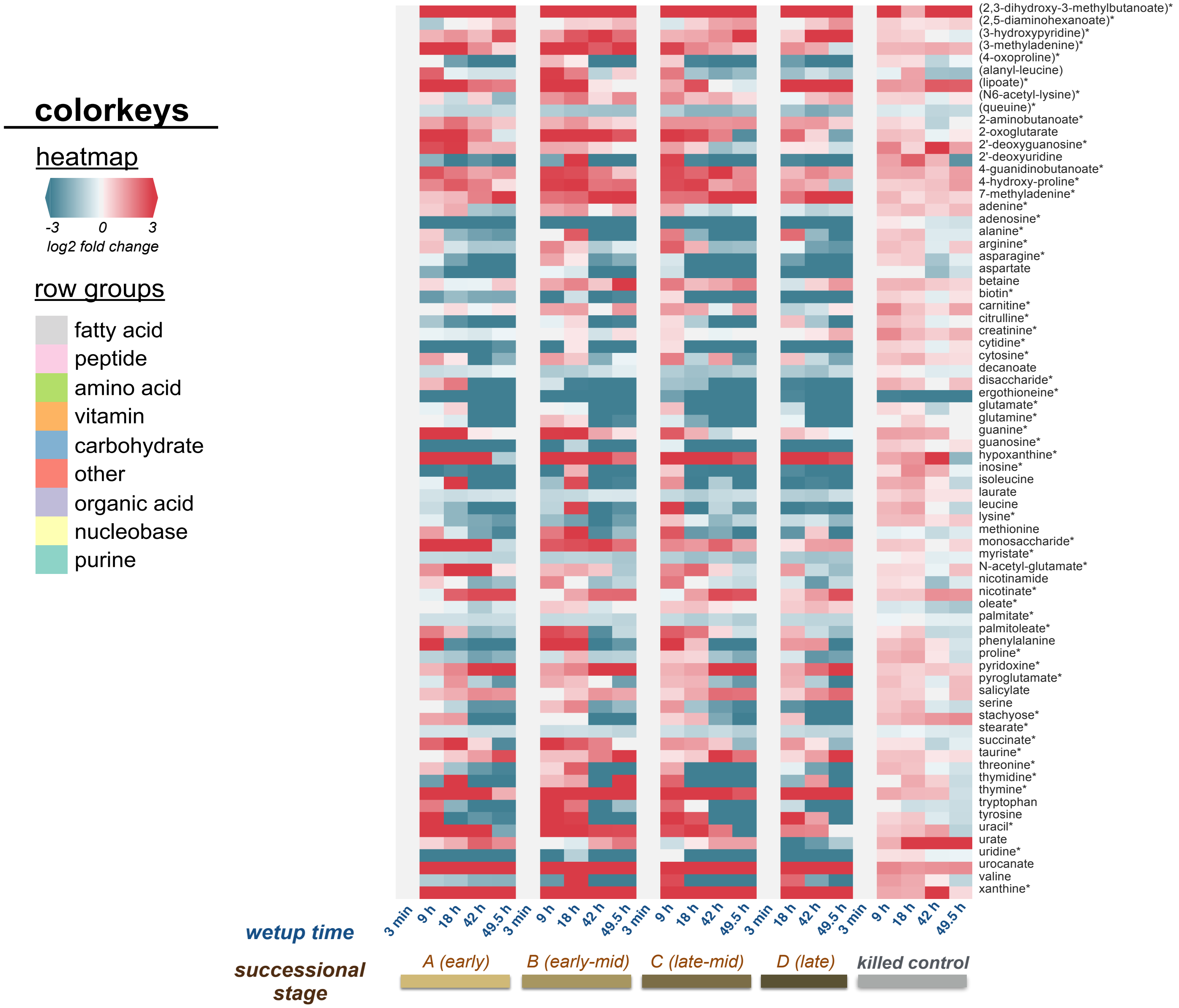

Supplementary Figure 3. Metabolite dynamics observed in biocrust soil water from active samples (early to late successional stages) compared to killed control samples. The heatmap displays changes in metabolite peak area (log2 fold change) relative to the first sampling timepoint (3 min) within each successional stage and within the killed control dataset for the 72 metabolites detected across all five datasets. Compared to active samples, 13 metabolites were not detected in the killed controls and therefore not shown. Putative metabolites are indicated by parentheses. n = 2-5 for each group. *$p < 0.05$ for metabolites that are significantly different in temporal patterns between the killed control and at least one successional stage of active biocrust based on two-way ANOVA and Tukey's post hoc test (Supplementary Data 3).

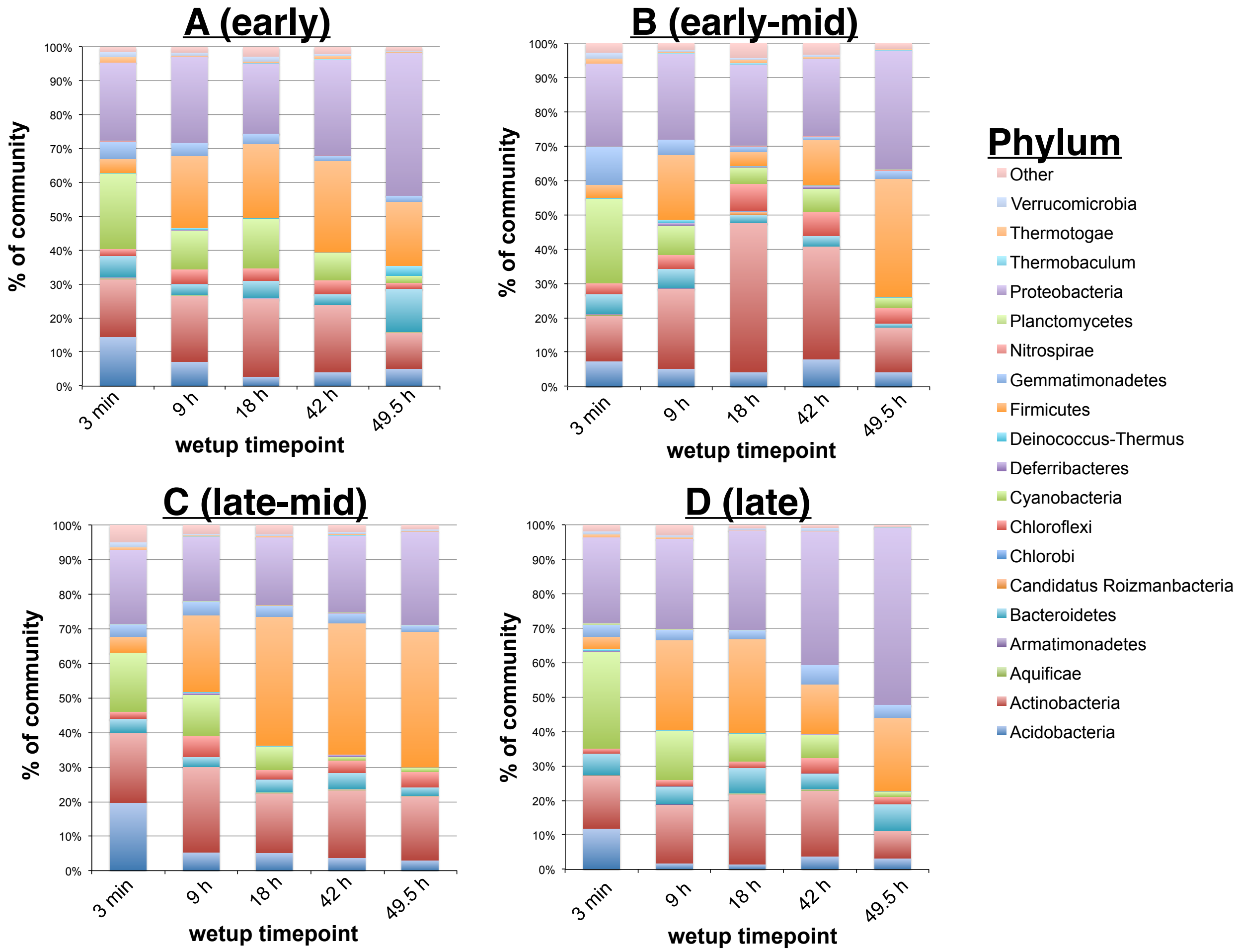

Supplementary Figure 4. Relative abundance of biocrust phyla across wetting for each successional stage A-D. n=1 for each group.

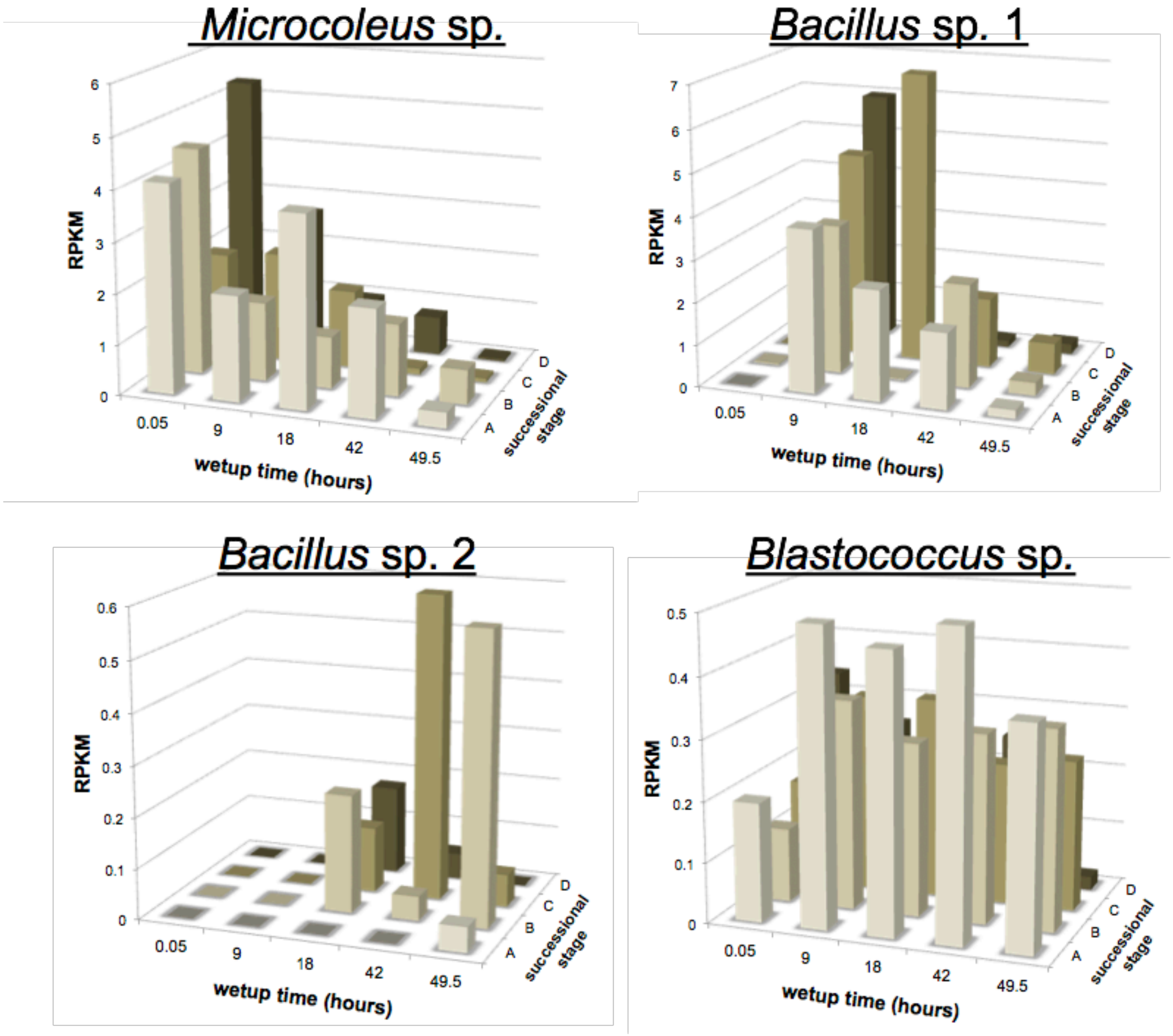

Supplementary Figure 5. Abundance distributions (estimated by RPKM values for the corresponding *rplO* gene) of the four isolate-related native biocrust bacteria across wetting and successional stages. n=1 for each group.

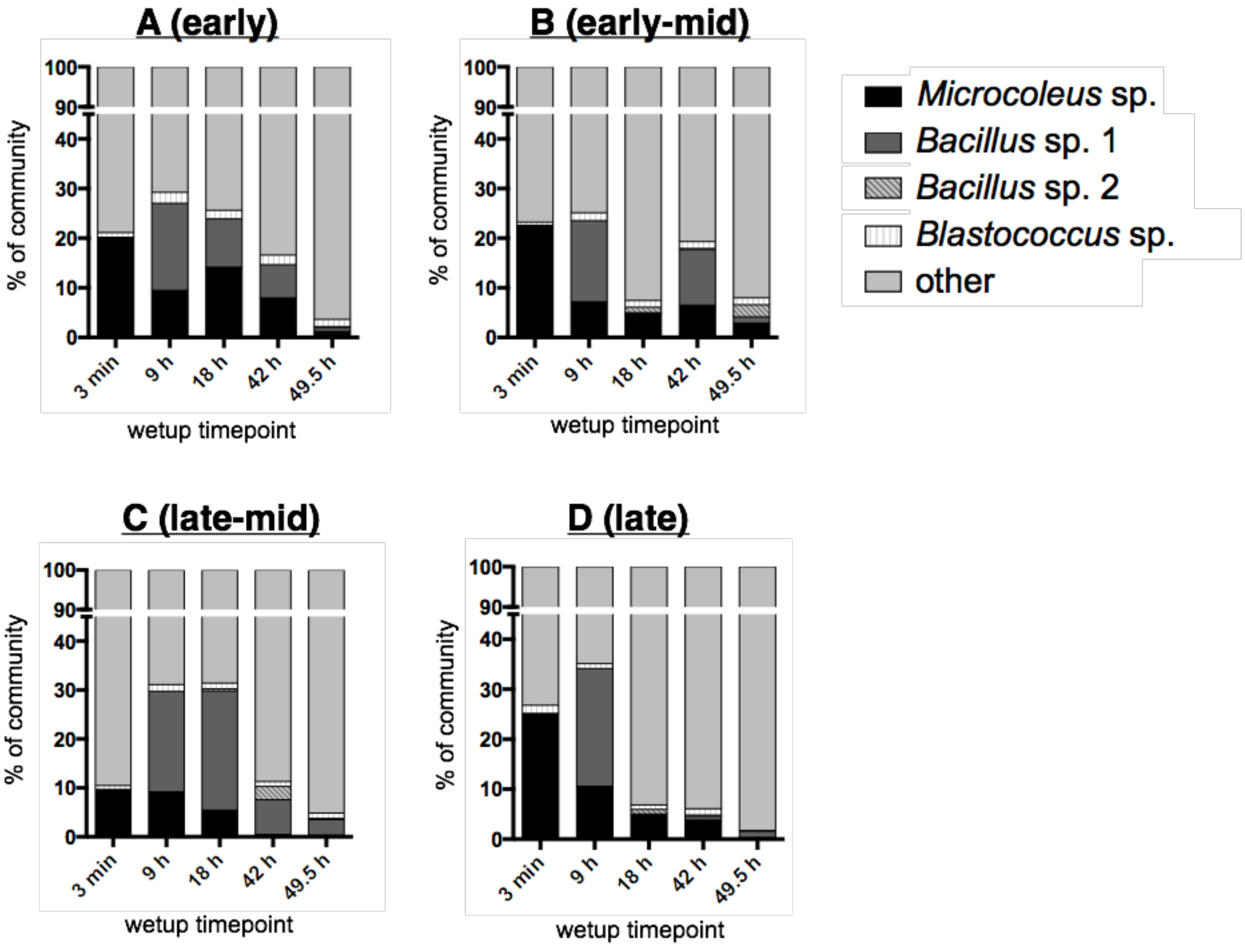

Supplementary Figure 6. Relative abundance of the four isolate-related native biocrust bacteria along wetting for each successional stage A-D. n=1 for each group.

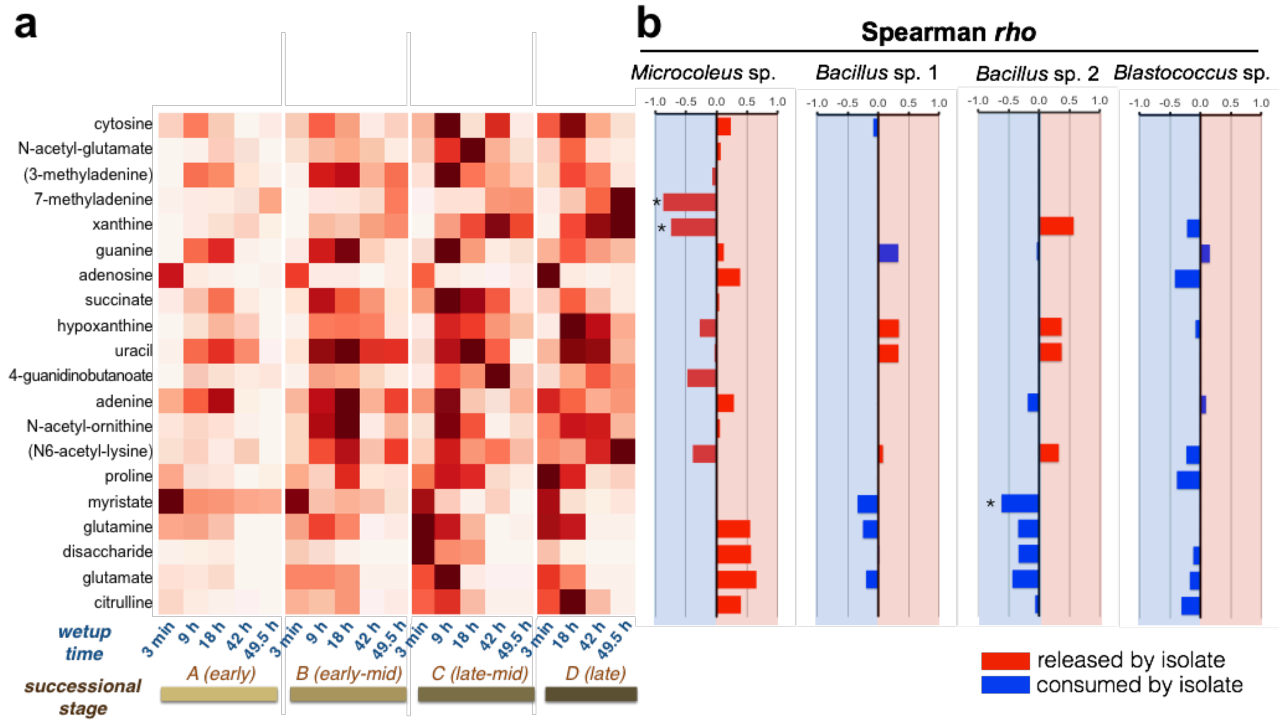

Supplementary Figure 7. Biocrust microbe-metabolite correlations of consumed and released metabolites. A. Abundance of exometabolite-related metabolites in the biocrust soil water after wetting and across successional stages. **B.** Spearman's *rho* correlations between each metabolite and the four relatively-abundant isolate-related native biocrust bacteria. Red bars are metabolites that were released by the related isolates while blue bars indicate consumed metabolites. * FDR-adjusted $p < 0.05$ for individual microbe-metabolite Spearman correlations.

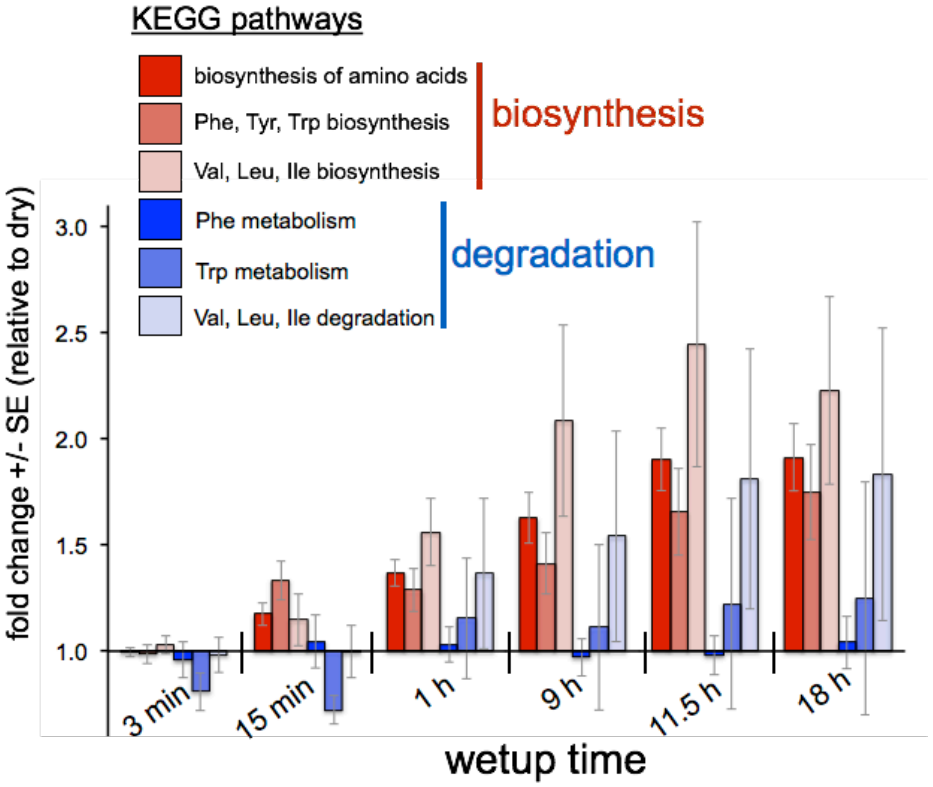

Supplementary Figure 8. Changes in *M. vaginatus* gene expression following wetup. The bar chart displays the average fold change and standard error of gene transcripts belonging to the indicated biosynthesis or degradation pathways along early wetup (3 min to 18 h) relative to dry biocrusts. Supplementary Data 8 lists the genes and fold-change for each KEGG pathway shown (data are from an earlier study of this same biocrust system; Rajeev et al[11]). n=1 for each time point.

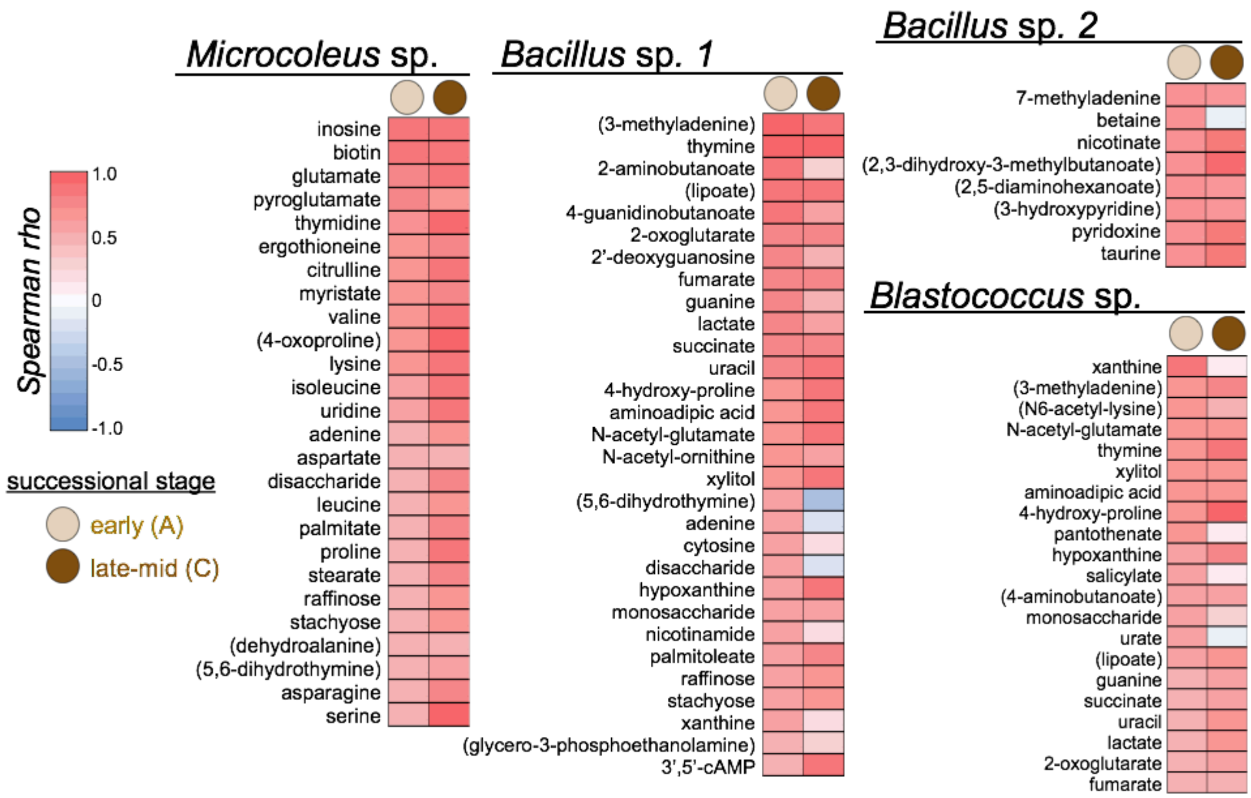

Supplementary Figure 9. Microbe-metabolite relationships in early versus mid-late successional stage biocrust. For the four bacteria *Microcoleus* sp., *Bacillus* sp. 1, *Bacillus* sp. 2 and *Blastococcus* sp., the most highly correlated (Spearman's *rho* ≥ 0.5) metabolites in early successional stage biocrust (level A) were analyzed for their degree of correlation in a later successional stage (level C) across wetting.

SUPPLEMENTARY TABLES

Supplementary Table 1. Ribosomal protein genes identified in biocrust metagenomes and their gene counts from the final assembly of all 20 samples.

gene	alternative name	gene counts
L2	rplB	434
L3	rplC	402
L4	rplD	436
L5	rplE	450
L6	rplF	447
L10	rplJ	430
L14	rplN	401
**L15**	**rplO**	**466**
L16	rplP	407
L18	rplR	401
L22	rplV	348
L24	rplX	425
S3	rpsC	375
S8	rpsH	461
S10	rpsJ	350
S17	rpsQ	412
S19	rpsS	355

Supplementary Table 2. Average amino acid identity (AAI) values between isolates and environmental genomes. AAI values were based on comparing coding loci between the two genomes of interest.

Biocrust Isolate	Closest Matching Biocrust Relative	AAI (%)
M. vaginatus PCC9802	*Microcoleus* sp. (*rplO* 1)	86.0
D1B51	*Bacillus* sp. 1 (*rplO* 2)	57.2
L2B47	*Bacillus* sp. 2 (*rplO* 60)	55.1
L1B44	*Blastococcus* sp. (*rplO* 7)	52.4

SUPPLEMENTARY REFERENCES

1. Langmead B, Salzberg SL. Fast gapped-read alignment with Bowtie 2. *Nature methods* **9:** 357-359 (2012).

2. Williams KH, Kemna A, Wilkins MJ, Druhan J, Arntzen E, N'Guessan AL *et al*. Geophysical monitoring of coupled microbial and geochemical processes during stimulated subsurface bioremediation. *Environmental Science & Technology* **43:** 6717-6723 (2009).

3. Alneberg J, Bjarnason BS, de Bruijn I, Schirmer M, Quick J, Ijaz UZ *et al*. Binning metagenomic contigs by coverage and composition. *Nature methods* **11:** 1144-1146 (2014).

4. Haft DH, Selengut JD, White O. The TIGRFAMs database of protein families. *Nucleic acids research* **31:** 371-373 (2003).

5. Finn RD, Bateman A, Clements J, Coggill P, Eberhardt RY, Eddy SR *et al*. Pfam: the protein families database. *Nucleic acids research* **42:** D222-230 (2014).

6. Hyatt D, Chen GL, Locascio PF, Land ML, Larimer FW, Hauser LJ. Prodigal: prokaryotic gene recognition and translation initiation site identification. *BMC bioinformatics* **11:** 119 (2010).

7. Edgar RC. Search and clustering orders of magnitude faster than BLAST. *Bioinformatics* **26:** 2460-2461 (2010).

8. Suzek BE, Wang Y, Huang H, McGarvey PB, Wu CH, UniProt C. UniRef clusters: a comprehensive and scalable alternative for improving sequence similarity searches. *Bioinformatics* **31:** 926-932 (2015).

9. Mulder N, Apweiler R. InterPro and InterProScan: tools for protein sequence classification and comparison. *Methods in molecular biology* **396:** 59-70 (2007).

10. Nawrocki EP, Eddy SR. Infernal 1.1: 100-fold faster RNA homology searches. *Bioinformatics* **29:** 2933-2935 (2013).

11. Rajeev, L. *et al.* Dynamic cyanobacterial response to hydration and dehydration in a desert biological soil crust. *ISME J* **7,** 2178–2191 (2013).
