# Supplementary material for: Linking soil biology and chemistry in biological soil crust using isolate exometabolomics
Source: Nat Commun. 2018 Jan 2;9:19. doi: 10.1038/s41467-017-02356-9 (PMC5750228; doi:10.1038/s41467-017-02356-9)
Supplement: Supplementary file 3 — Description of Additional Supplementary Files [file 41467_2017_2356_MOESM3_ESM.pdf]

Description of Additional Supplementary Files

File Name: Supplementary Data 1
Description: Metabolites detected in biocrust soil water.

File Name: Supplementary Data 2
Description: Metabolites detected in killed control biocrust samples.

File Name: Supplementary Data 3
Description: ANOVA results for killed control vs. active biocrust from 4 successional stages. A "1" in columns E-H indicate $p < 0.05$ (temporal patterns were significantly different)

File Name: Supplementary Data 4
Description: Distinct rplO genes identified and their corresponding taxonomic information and rpkm values across samples.

File Name: Supplementary Data 5
Description: Comparison of 16 ribosomal protein gene phylogenetic markers (between four biocrust metagenome-assembled genomes and their closest related isolates).

File Name: Supplementary Data 6
Description: Isolate exometabolomics versus biocrust soil water (metabolites whose temporal patterns were not significantly different from the killed controls were excluded).

File Name: Supplementary Data 7
Description: Binomial test of microbe-metabolite relationships

File Name: Supplementary Data 8
Description: Data obtained from Rajeev et al (2013) and categorized into relevant KEGG pathways. Values indicate fold change relative to dry biocrust (time 0).
